# Supplementary material for: Pyruvate Dehydrogenase Kinase 1 inhibition mediated oxidative phosphorylation enhancement in cartilage promotes osteoarthritis progression
Source: BMC Musculoskelet Disord. 2023 Jul 20;24:597. doi: 10.1186/s12891-023-06585-6 (PMC10357736; doi:10.1186/s12891-023-06585-6)
Supplement: Supplementary file 1 — Additional file 1. [file 12891_2023_6585_MOESM1_ESM.zip › MF.Gsea.1653627316242/GOMF_SMAD_BINDING.html]

Details for gene set GOMF\_SMAD\_BINDING[GSEA]

|  || Dataset | 55457\_collapsed\_to\_symbols.cls.cls #OA\_versus\_normal.cls.cls #OA\_versus\_normal\_repos |
| Phenotype | cls.cls#OA\_versus\_normal\_repos |
| Upregulated in class | normal |
| GeneSet | GOMF\_SMAD\_BINDING |
| Enrichment Score (ES) | -0.39143464 |
| Normalized Enrichment Score (NES) | -1.6225189 |
| Nominal p-value | 0.007936508 |
| FDR q-value | 0.13776962 |
| FWER p-Value | 0.988 |
Table: GSEA Results Summary

  

Fig 1: Enrichment plot: GOMF\_SMAD\_BINDING      
 Profile of the Running ES Score & Positions of GeneSet Members on the Rank Ordered List

  

| SYMBOL | TITLE | RANK IN GENE LIST | RANK METRIC SCORE | RUNNING ES | CORE ENRICHMENT || 1 | SMAD2 | SMAD family member 2 [Source:HGNC Symbol;Acc:HGNC:6768] | 275 | 0.768 | 0.0120 | No |
| 2 | PAX6 | paired box 6 [Source:HGNC Symbol;Acc:HGNC:8620] | 304 | 0.746 | 0.0423 | No |
| 3 | STUB1 | STIP1 homology and U-box containing protein 1 [Source:HGNC Symbol;Acc:HGNC:11427] | 345 | 0.726 | 0.0708 | No |
| 4 | LDLRAD4 | low density lipoprotein receptor class A domain containing 4 [Source:HGNC Symbol;Acc:HGNC:1224] | 879 | 0.548 | 0.0531 | No |
| 5 | DAB2 | DAB adaptor protein 2 [Source:HGNC Symbol;Acc:HGNC:2662] | 1821 | 0.381 | -0.0036 | No |
| 6 | MAGI2 | "membrane associated guanylate kinase, WW and PDZ domain containing 2 [Source:HGNC Symbol;Acc:HGNC:18957]" | 1980 | 0.363 | -0.0002 | No |
| 7 | DROSHA | drosha ribonuclease III [Source:HGNC Symbol;Acc:HGNC:17904] | 2054 | 0.356 | 0.0096 | No |
| 8 | FKBP1A | FKBP prolyl isomerase 1A [Source:HGNC Symbol;Acc:HGNC:3711] | 2293 | 0.328 | 0.0053 | No |
| 9 | BMPR1A | bone morphogenetic protein receptor type 1A [Source:HGNC Symbol;Acc:HGNC:1076] | 2745 | 0.275 | -0.0178 | No |
| 10 | GATA4 | GATA binding protein 4 [Source:HGNC Symbol;Acc:HGNC:4173] | 3009 | 0.249 | -0.0275 | No |
| 11 | PARP1 | poly(ADP-ribose) polymerase 1 [Source:HGNC Symbol;Acc:HGNC:270] | 3410 | 0.210 | -0.0495 | No |
| 12 | SMAD6 | SMAD family member 6 [Source:HGNC Symbol;Acc:HGNC:6772] | 3460 | 0.206 | -0.0444 | No |
| 13 | TGFB1I1 | transforming growth factor beta 1 induced transcript 1 [Source:HGNC Symbol;Acc:HGNC:11767] | 3628 | 0.191 | -0.0491 | No |
| 14 | ACVR1 | activin A receptor type 1 [Source:HGNC Symbol;Acc:HGNC:171] | 3898 | 0.165 | -0.0629 | No |
| 15 | SMAD3 | SMAD family member 3 [Source:HGNC Symbol;Acc:HGNC:6769] | 4061 | 0.150 | -0.0690 | No |
| 16 | PURA | purine rich element binding protein A [Source:HGNC Symbol;Acc:HGNC:9701] | 4514 | 0.112 | -0.0993 | No |
| 17 | COL5A2 | collagen type V alpha 2 chain [Source:HGNC Symbol;Acc:HGNC:2210] | 5262 | 0.051 | -0.1553 | No |
| 18 | TGFBR1 | transforming growth factor beta receptor 1 [Source:HGNC Symbol;Acc:HGNC:11772] | 5280 | 0.050 | -0.1545 | No |
| 19 | SNW1 | SNW domain containing 1 [Source:HGNC Symbol;Acc:HGNC:16696] | 5472 | 0.035 | -0.1679 | No |
| 20 | MEF2A | myocyte enhancer factor 2A [Source:HGNC Symbol;Acc:HGNC:6993] | 5628 | 0.022 | -0.1790 | No |
| 21 | RNF111 | ring finger protein 111 [Source:HGNC Symbol;Acc:HGNC:17384] | 5711 | 0.015 | -0.1848 | No |
| 22 | BMPR1B | bone morphogenetic protein receptor type 1B [Source:HGNC Symbol;Acc:HGNC:1077] | 5959 | -0.002 | -0.2039 | No |
| 23 | PPARG | peroxisome proliferator activated receptor gamma [Source:HGNC Symbol;Acc:HGNC:9236] | 6006 | -0.005 | -0.2073 | No |
| 24 | ZMIZ1 | zinc finger MIZ-type containing 1 [Source:HGNC Symbol;Acc:HGNC:16493] | 6111 | -0.013 | -0.2148 | No |
| 25 | ACVR1B | activin A receptor type 1B [Source:HGNC Symbol;Acc:HGNC:172] | 6368 | -0.033 | -0.2334 | No |
| 26 | TCF12 | transcription factor 12 [Source:HGNC Symbol;Acc:HGNC:11623] | 6398 | -0.035 | -0.2341 | No |
| 27 | ANKRD1 | ankyrin repeat domain 1 [Source:HGNC Symbol;Acc:HGNC:15819] | 6416 | -0.037 | -0.2338 | No |
| 28 | PML | PML nuclear body scaffold [Source:HGNC Symbol;Acc:HGNC:9113] | 6421 | -0.037 | -0.2325 | No |
| 29 | SMAD5 | SMAD family member 5 [Source:HGNC Symbol;Acc:HGNC:6771] | 6530 | -0.045 | -0.2390 | No |
| 30 | USP9X | ubiquitin specific peptidase 9 X-linked [Source:HGNC Symbol;Acc:HGNC:12632] | 6642 | -0.053 | -0.2454 | No |
| 31 | HMGA2 | high mobility group AT-hook 2 [Source:HGNC Symbol;Acc:HGNC:5009] | 6850 | -0.067 | -0.2586 | No |
| 32 | TGFBR3 | transforming growth factor beta receptor 3 [Source:HGNC Symbol;Acc:HGNC:11774] | 6868 | -0.069 | -0.2569 | No |
| 33 | RANBP3 | RAN binding protein 3 [Source:HGNC Symbol;Acc:HGNC:9850] | 7043 | -0.083 | -0.2669 | No |
| 34 | CITED2 | Cbp/p300 interacting transactivator with Glu/Asp rich carboxy-terminal domain 2 [Source:HGNC Symbol;Acc:HGNC:1987] | 7100 | -0.087 | -0.2674 | No |
| 35 | SMAD7 | SMAD family member 7 [Source:HGNC Symbol;Acc:HGNC:6773] | 7231 | -0.098 | -0.2733 | No |
| 36 | CREB3L1 | cAMP responsive element binding protein 3 like 1 [Source:HGNC Symbol;Acc:HGNC:18856] | 7324 | -0.104 | -0.2760 | No |
| 37 | TGFBRAP1 | transforming growth factor beta receptor associated protein 1 [Source:HGNC Symbol;Acc:HGNC:16836] | 7347 | -0.106 | -0.2731 | No |
| 38 | SKI | SKI proto-oncogene [Source:HGNC Symbol;Acc:HGNC:10896] | 7349 | -0.106 | -0.2686 | No |
| 39 | MEN1 | menin 1 [Source:HGNC Symbol;Acc:HGNC:7010] | 7542 | -0.122 | -0.2782 | No |
| 40 | TOB1 | "transducer of ERBB2, 1 [Source:HGNC Symbol;Acc:HGNC:11979]" | 7892 | -0.147 | -0.2990 | No |
| 41 | PPM1A | "protein phosphatase, Mg2+/Mn2+ dependent 1A [Source:HGNC Symbol;Acc:HGNC:9275]" | 8125 | -0.165 | -0.3099 | No |
| 42 | TGFBR2 | transforming growth factor beta receptor 2 [Source:HGNC Symbol;Acc:HGNC:11773] | 8241 | -0.174 | -0.3113 | No |
| 43 | USP15 | ubiquitin specific peptidase 15 [Source:HGNC Symbol;Acc:HGNC:12613] | 8653 | -0.205 | -0.3345 | No |
| 44 | COL3A1 | collagen type III alpha 1 chain [Source:HGNC Symbol;Acc:HGNC:2201] | 8784 | -0.215 | -0.3352 | No |
| 45 | SAV1 | salvador family WW domain containing protein 1 [Source:HGNC Symbol;Acc:HGNC:17795] | 8960 | -0.229 | -0.3389 | No |
| 46 | SMAD4 | SMAD family member 4 [Source:HGNC Symbol;Acc:HGNC:6770] | 9035 | -0.235 | -0.3345 | No |
| 47 | FERMT2 | FERM domain containing kindlin 2 [Source:HGNC Symbol;Acc:HGNC:15767] | 9349 | -0.259 | -0.3476 | No |
| 48 | FOXH1 | forkhead box H1 [Source:HGNC Symbol;Acc:HGNC:3814] | 9884 | -0.310 | -0.3757 | Yes |
| 49 | SMAD1 | SMAD family member 1 [Source:HGNC Symbol;Acc:HGNC:6767] | 9905 | -0.312 | -0.3637 | Yes |
| 50 | COL1A2 | collagen type I alpha 2 chain [Source:HGNC Symbol;Acc:HGNC:2198] | 9985 | -0.320 | -0.3560 | Yes |
| 51 | AXIN1 | axin 1 [Source:HGNC Symbol;Acc:HGNC:903] | 10335 | -0.354 | -0.3677 | Yes |
| 52 | FOS | "Fos proto-oncogene, AP-1 transcription factor subunit [Source:HGNC Symbol;Acc:HGNC:3796]" | 10640 | -0.387 | -0.3746 | Yes |
| 53 | IPO7 | importin 7 [Source:HGNC Symbol;Acc:HGNC:9852] | 10662 | -0.389 | -0.3593 | Yes |
| 54 | BMP2 | bone morphogenetic protein 2 [Source:HGNC Symbol;Acc:HGNC:1069] | 10919 | -0.420 | -0.3610 | Yes |
| 55 | PMEPA1 | "prostate transmembrane protein, androgen induced 1 [Source:HGNC Symbol;Acc:HGNC:14107]" | 11024 | -0.433 | -0.3502 | Yes |
| 56 | SKIL | SKI like proto-oncogene [Source:HGNC Symbol;Acc:HGNC:10897] | 11253 | -0.464 | -0.3478 | Yes |
| 57 | ZC3H3 | zinc finger CCCH-type containing 3 [Source:HGNC Symbol;Acc:HGNC:28972] | 11571 | -0.511 | -0.3503 | Yes |
| 58 | ZBTB7A | zinc finger and BTB domain containing 7A [Source:HGNC Symbol;Acc:HGNC:18078] | 11600 | -0.516 | -0.3300 | Yes |
| 59 | SMAD9 | SMAD family member 9 [Source:HGNC Symbol;Acc:HGNC:6774] | 11633 | -0.522 | -0.3098 | Yes |
| 60 | TRIM33 | tripartite motif containing 33 [Source:HGNC Symbol;Acc:HGNC:16290] | 11666 | -0.527 | -0.2893 | Yes |
| 61 | SMURF2 | SMAD specific E3 ubiquitin protein ligase 2 [Source:HGNC Symbol;Acc:HGNC:16809] | 11802 | -0.550 | -0.2759 | Yes |
| 62 | USP9Y | ubiquitin specific peptidase 9 Y-linked [Source:HGNC Symbol;Acc:HGNC:12633] | 12058 | -0.609 | -0.2693 | Yes |
| 63 | YY1 | YY1 transcription factor [Source:HGNC Symbol;Acc:HGNC:12856] | 12229 | -0.653 | -0.2541 | Yes |
| 64 | ACVRL1 | activin A receptor like type 1 [Source:HGNC Symbol;Acc:HGNC:175] | 12242 | -0.656 | -0.2265 | Yes |
| 65 | SMURF1 | SMAD specific E3 ubiquitin protein ligase 1 [Source:HGNC Symbol;Acc:HGNC:16807] | 12344 | -0.688 | -0.2044 | Yes |
| 66 | RGCC | regulator of cell cycle [Source:HGNC Symbol;Acc:HGNC:20369] | 12478 | -0.740 | -0.1826 | Yes |
| 67 | CITED1 | Cbp/p300 interacting transactivator with Glu/Asp rich carboxy-terminal domain 1 [Source:HGNC Symbol;Acc:HGNC:1986] | 12493 | -0.746 | -0.1512 | Yes |
| 68 | JUN | "Jun proto-oncogene, AP-1 transcription factor subunit [Source:HGNC Symbol;Acc:HGNC:6204]" | 12813 | -0.994 | -0.1328 | Yes |
| 69 | DDX5 | DEAD-box helicase 5 [Source:HGNC Symbol;Acc:HGNC:2746] | 12834 | -1.038 | -0.0892 | Yes |
| 70 | HIPK2 | homeodomain interacting protein kinase 2 [Source:HGNC Symbol;Acc:HGNC:14402] | 12842 | -1.045 | -0.0443 | Yes |
| 71 | CTNNB1 | catenin beta 1 [Source:HGNC Symbol;Acc:HGNC:2514] | 12866 | -1.119 | 0.0026 | Yes |
Table: GSEA details [plain text format]

  

Fig 2: GOMF\_SMAD\_BINDING      
 Blue-Pink O' Gram in the Space of the Analyzed GeneSet

  

Fig 3: GOMF\_SMAD\_BINDING: Random ES distribution      
 Gene set null distribution of ES for **GOMF\_SMAD\_BINDING**
